# Global burden and trends of pelvic inflammatory disease associated with sexually transmitted infection excluding HIV from 1990 to 2021

**DOI:** 10.3389/fgwh.2025.1658086

**Published:** 2025-12-16

**Authors:** Jie Li, Tianyu Li, Zhong Lin, Hui Li, Zengnan Mo, Jinling Liao, Yang Chen

**Affiliations:** 1 The Reproductive Hospital of Guangxi Zhuang Autonomous Region, Nanning, Guangxi, China; 2 Center for Genomic and Personalized Medicine, Guangxi Key Laboratory for Genomic and Personalized Medicine, Guangxi Collaborative Innovation Center for Genomic and Personalized Medicine, Guangxi Medical University, Nanning, Guangxi, China; 3 Department of Urology, The First Affiliated Hospital of Guangxi Medical University, Nanning, Guangxi, China

**Keywords:** pelvic inflammatory disease, GBD database, non-HIV, EAPC, SDI

## Abstract

**Background:**

Pelvic inflammatory disease (PID) is mainly induced by the sexually transmitted infection (STI). However, the global burden and trends of STI excluding human immunodeficiency virus (HIV)-associated PID (non-HIV PID) has not been specifically assessed.

**Methods:**

The prevalence and years lived with disability (YLDs) were collected from the Global Burden of Disease (GBD) 2021 database. The disease burden was evaluated with the case numbers, age-standardized rates (ASR) and estimated annual percentage changes (EAPC). According to the SocioDemographic Index (SDI), frontier and health inequality analysis were conducted. Autoregressive Integrated Moving Average (ARIMA) model was applied to predict the future trends of Non-HIV PID.

**Results:**

The age-standardized prevalence rates (ASPR) and YLDs of non-HIV PID was 27.02/100,000 and 3.68/100,000 in 2021 globally. Except for the decline of gonococcal-associated PID, the EAPC of chlamydial and other non-HIV PID were stable. The countries with fastest-growing prevalence were Brazil (4.19 [2.92, 5.47]), Spain (3.98 [3.19, 4.77]), Greece (3.05 [2.55, 3.55]), Portugal (2.76 [2.22, 3.29]), which suggested the increased burden of non-HIV PID in these years. Moreover, the non-HIV PID was mainly concentrated in 30–34 years, which was most common in the low and low-middle SDI. Additionally, prevention of non-HIV PID should also be concerned in the high SDI regions, especially for United Kingdom, Canada, Japan, and Singapore, which would also increase in the next 30 years.

**Conclusion:**

The burden and prevention of non-HIV PID were still arduous and required a long-term effort, especially for the 30–34 years, which need more attentions even for the developed countries.

## Introduction

Pelvic inflammatory disease (PID) is a female upper reproductive tract disease. The diagnosis is mainly based on the clinical symptoms or laparoscopy. Although laparoscopy had high sensitivity in diagnosing the PID, the invasive procedure was unpractical for the primary care (1). So, the definite incidence rate of PID is unclear because of the subtle or even absent symptoms (2). Some study estimated the rate of PID diagnosis was about 280/100,000 person years, mainly influencing the women aged 20–29 (3). The epidemiology from America identified the self-reported history of PID in women aged 18–44 years was about 4% (4, 5). PID endangered the female reproductive health directly, especially during the childbearing age. Long-term follow-up discovered the PID would induce the infertility, ectopic pregnancy and chronic pelvic pain even after antibiotic treatment (6, 7). Moreover, the delaying treatments after the symptoms of PID would increase the risk of post-PID infertility or ectopic pregnancy for more than three times (8, 9). So, grasping the PID incidence situation would be important in the deployment of health strategies.

PID is mainly induced by the microbial pathogens, especially for the sexually transmitted infections (STIs), such as the Chlamydia trachomatis, Neisseria gonorrhoeae, Mycoplasma genitalium, endogenous vaginal organisms (Bacteroides species) and human immunodeficiency virus (HIV) (2). Among the STIs, more efforts and priorities had been put on the HIV previously, comparing to non-HIV diseases (10). However, the incidence of non-HIV STIs also accounted for a certain high proportion, which should also need to receive the same priority as HIV (16). Moreover, approximate 50% of women presented with PID were induced by the non-HIV STIs, such as Chlamydia trachomatis and Neisseria gonorrhea (11). Even so, the actual prevalence especially for the non-HIV PID was still unclear (6). This time, we firstly used the Global Burden of Disease (GBD) 2021 database to analyze the burden of non-HIV PID all over the world. The results not only presented the global distributions of non-HIV PID, but also forecasted the prevalence and years lived with disability (YLDs) tendency in the next 30 years, which would pave a way for the personalized preventive care and treatment in different regions and countries.

## Methods and materials

### GBD database

The data was collected from the GBD 2021 database, which was mainly focused on the worldwide estimates of various disease burdens (12, 13). The project was initiated by the Institute for Health Metrics and Evaluation (IHME), containing the essential crowd information, such as age, sex, year and geographic region. Moreover, it also recorded the epidemiologic features of diseases, including incidence, prevalence, mortality, years of life lost (YLLs), YLDs, disability-adjusted life years (DALYs), etc. The indexes of GBD estimates included the Causes of death or injury, Risk factor, Etiology, Impairment, etc. And the metrics of the data were Number, Rate, Percent, etc., which were presented with their 95% uncertainty intervals (UIs). All the details were presented on the IHME website (https://VizHub.healthdata.org/gbd-results/).

### Data acquisition

In order to discuss the prevalence of PID caused by non-HIV STI, the prevalence and YLDs of PID, moderate PID and severe PID were downloaded from the GBD 2021 database. However, there were no explicit definitions for the moderate and severe PID. So, in this study, fewer analyses were performed for these two groups. In the study, the causes included the chlamydia, gonococcal and other STIs from 204 countries, territories and 811 subnational locations. The HIV co-infection was also excluded from the analysis. All ages, age-standardized and 5-year intervals (<5 years, 5–9 years, 10–14 years, 15–19 years, 20–24 years, 25–29 years, 30–34 years, 35–39 years, 40–44 years, 45–49 years, 50–54 years, 55–59 years, 60–64 years, 65–69 years, 70–74 years, 75–79 years, 80–84 years, 85–89 years, ≥90 years) were recorded in the analysis from 1990 to 2021. Additionally, the SocioDemographic Index (SDI) was applied to evaluate the socioeconomic effects in the non-HIV PID in all the countries (14).

### Statistical analysis

Estimated Annual Percentage Change (EAPC) of rate for the age-standardized prevalence (ASPR) and YLDs were calculated from 1990 to 2021 for all the regions and countries. In order to assess the fluctuation of the prevalence and YLDs trends, the Joinpoint software (5.4.0) was used to perform the piecewise regression analysis with the visual trend of disease changes, providing a basis for the subsequent formulation of medical policies and resource allocation. The Loglinear model was selected in the Joinpoint analysis, with the max joinpoints set as 5. And the others follow the default parameters. Then, frontier analysis and health inequality analysis were applied to explore the relationship between the SDI of each country and the disease control situation, by measuring the disease prevalence and YLDs. Finally, Autoregressive Integrated Moving Average (ARIMA) model in forecast R package was also employed to predict the trend of disease occurrence. ARIMA combines the autoregressive (AR) and moving average (MA) methods and incorporates the concept of differencing (I) to make non-stationary time series data stationary, thereby enabling effective forecasting. The values of Akaike Information Criterion/Bayesian Information Criterion (AIC/BIC) and 95% confidence intervals (CI) were used to evaluate the fitting status of the model and statistical reliability. All analyses were conducted with R software (4.4.2). *P* < 0.05 was treated as statistical significance.

## Results

### Global situation of Non-HIV PID

In order to display the prevalence and YLDs of PID correlated to the non-HIV, the global data were analyzed in the GBD super regions firstly. From 1990 to 2021, the numbers of prevalent and YLDs of non-HIV PID increased from 6645.17 × 10^2^ (95% UI: 5,090.93–8,472.08) to 10,895.44 × 10^2^ (95% UI: 8,151.64–14,055.2), and 904.3 * 10^2^ (95% UI: 566.54–1,370.65) to 1,484.07 × 10^2^ (95% UI: 915.12–2,269.91) globally. However, both the EAPC of ASPR and YLDs did not reach statistical significance (−0.04 [−0.09 to 0.01]), which suggested the stable disease burden by controlling STIs. While the regions were divided into five groups according to the SDI, the slight increases of EAPC in prevalence and YLDs presented in the high-middle SDI (0.25 [0.2–0.31]) and middle SDI (0.37 [0.34–0.41]). And they decreased in the low-middle SDI and low SDI regions. (Table 1) Additionally, among GBD super regions, the EAPC of ASPR (1.46 [0.99–1.93]) or YLDs (1.45 [0.99–1.92]) were significant increase in the Latin America and Caribbean. And the noteworthy decline of EAPC of ASPR (−2.07 [−2.39 to −1.76]) or YLDs (−2.06 [−2.37 to −1.74]) presented in the Sahel Region (Table 1).

**Table 1:** The prevalence and years lived with disability (YLDs) of pelvic inflammatory disease correlated to sexually transmitted infections excluding HIV in the GBD super regions. ASPR: age-standardized prevalence rates; EAPC: estimated annual percentage change.

Location	1990				2021				1990–2021	
	Prevalent cases No. *102 (95% UI)	ASPR per 100,000 No. (95% UI)	YLD cases No. *102 (95% UI)	Age-standardized YLD rate per 100,000 No. (95% UI)	Prevalent cases No. *102 (95% UI)	ASPR per 100,000 No.(95% UI)	YLD cases No. *102 (95% UI)	Age-standardized YLD rate per 100,000 No.(95% UI)	EAPC of ASPR No. (95% CI)	EAPC of age-standardized YLD rate No. (95% CI)
Global	6,645.17 [5,090.93–8,472.08]	25.82 [19.66–32.82]	904.3 [566.54–1,370.65]	3.51 [2.2–5.33]	10,895.44 [8,151.64–14,055.2]	27.02 [20.24–34.89]	1,484.07 [915.12–2,269.91]	3.68 [2.27–5.63]	−0.04 [−0.09 to 0.01]	−0.04 [−0.09 to 0.01]
Socio-demographic index										
High SDI	1,122.21 [830.65–1,468.58]	23.7 [17.53–30.99]	153.9 [90.22–236.01]	3.25 [1.91–4.96]	1,336.21 [1,032.44–1,695.94]	25.12 [19.43–31.49]	182.94 [114.71–274.15]	3.44 [2.17–5.14]	−0.08 [−0.19 to 0.02]	−0.08 [−0.19 to 0.02]
High-middle SDI	1,101.09 [817.91–1,444.42]	19.95 [14.81–26.22]	150.52 [90.61–229.43]	2.73 [1.64–4.18]	1,485.89 [1,116.53–1,926.75]	21.01 [15.8–27.25]	202.87 [123.85–312.36]	2.87 [1.74–4.43]	0.25 [0.2 to 0.31]	0.25 [0.2 to 0.31]
Middle SDI	1,660.51 [1,235.32–2,149.47]	20.35 [15.26–26.33]	226.45 [139.98–347.07]	2.77 [1.71–4.21]	3,076.65 [2,314.12–3,962.19]	23.29 [17.49–29.84]	419.27 [255.05–640.9]	3.17 [1.93–4.84]	0.37 [0.34 to 0.41]	0.37 [0.34 to 0.41]
Low-middle SDI	1,695.98 [1,329.46–2,156.27]	33.52 [26.41–42.1]	229.46 [147.34–345.08]	4.53 [2.89–6.84]	3,051.63 [2,267.89–4,036.38]	30.72 [22.9–40.6]	415.09 [250.75–637.53]	4.18 [2.52–6.42]	−0.55 [–0.65 to −0.45]	−0.53 [−0.64 to −0.43]
Low SDI	1,059.83 [843.61–1,324.06]	50.35 [40.43–62.22]	143.22 [93.73–216.41]	6.8 [4.42–10.22]	1,938.46 [1,423.77–2,586.62]	38.23 [28.28–50.35]	262.99 [159.57–402.37]	5.19 [3.13–7.9]	−1.39 [−1.59 to −1.18]	−1.37 [−1.57 to −1.17]
Region										
Southeast Asia, East Asia, and Oceania	1,351.04 [999.1–1,783.1]	16.33 [12.07–21.58]	184.75 [111.44–284.34]	2.23 [1.35–3.46]	1,970.98 [1,494.13–2,531.02]	16.48 [12.53–21.36]	268.91 [165.26–413.7]	2.25 [1.37–3.48]	0.11 [−0.01 to 0.24]	0.11 [−0.01 to 0.23]
North Africa and Middle East	238.87 [176.11–312.21]	17.05 [12.68–22.24]	32.62 [19.4–50.19]	2.33 [1.41–3.54]	557.55 [406.65–737.75]	17.18 [12.68–22.59]	76.25 [45.18–118.75]	2.35 [1.39–3.66]	0.24 [0.14 to 0.34]	0.25 [0.14 to 0.35]
Central Europe, Eastern Europe, and Central Asia	576.43 [430.82–750.54]	26.22 [19.61–34.15]	78.63 [47.26–119.94]	3.58 [2.15–5.44]	576.5 [429.58–750.77]	25.97 [19.44–33.78]	78.72 [47.61–122.67]	3.55 [2.16–5.47]	−0.09 [−0.18 to 0]	−0.09 [−0.18 to 0]
Latin America and Caribbean	364.54 [277.12–466.16]	19.64 [14.88–25.02]	49.84 [30.74–74.82]	2.69 [1.68–4.06]	957 [734.64–1,220.94]	29.25 [22.42–37.25]	130.61 [81.13–198.43]	3.99 [2.49–6.05]	1.46 [0.99 to 1.93]	1.45 [0.99 to 1.92]
High-income	1,120.91 [832.27–1,468.84]	23.13 [17.14–30.22]	153.76 [90.63–234.01]	3.17 [1.87–4.83]	1,329.12 [1,028.66–1,677.05]	25.72 [19.85–32.28]	182.08 [114.19–271.26]	3.52 [2.21–5.29]	0.09 [−0.03 to 0.21]	0.09 [−0.03 to 0.21]
South Asia	1,742.91 [1,369.2–2,223.2]	36.6 [28.8–46.33]	235.63 [148.34–358.09]	4.95 [3.15–7.48]	3,223.93 [2,380.74–4,292.34]	33.13 [24.62–44.24]	438.05 [260.86–676.85]	4.5 [2.68–6.97]	−0.59 [−0.7 to −0.48]	−0.58 [−0.68 to −0.47]
Sub-Saharan Africa	1,250.48 [973.44–1,580.29]	58.75 [46.26–73.86]	169.07 [108.77–256.09]	7.94 [5.18–12.11]	2,280.36 [1,655.05–3,116.95]	42.94 [31.02–57.6]	309.46 [188.58–480.21]	5.83 [3.54–9.05]	−1.64 [−1.86 to −1.42]	−1.63 [−1.85 to −1.41]
WHO region	6,616.72 [5,070.06–8,435.17]	25.86 [19.7–32.88]	900.41 [564.23–1,364.6]	3.52 [2.2–5.34]	10,861.63 [8,126.08–14,012.04]	27.05 [20.27–34.93]	1,479.43 [912.26–2,262.23]	3.69 [2.27–5.63]	−0.05 [−0.1 to 0]	−0.04 [−0.09 to 0.01]
European Union	268.68 [196.49–351.73]	12.22 [8.95–16.17]	36.83 [21.88–56.29]	1.68 [0.98–2.56]	302.07 [234.58–378.71]	14.28 [11.05–17.89]	41.35 [25.87–61.26]	1.95 [1.23–2.96]	0.68 [0.48 to 0.88]	0.67 [0.47 to 0.87]
World Bank Regions	6,637.35 [5,084.87–8,462.2]	25.82 [19.67–32.83]	903.24 [565.86–1,369.01]	3.51 [2.2–5.33]	10,886.15 [8,144.68–14,043.3]	27.02 [20.24–34.89]	1,482.8 [914.31–2,268.04]	3.68 [2.27–5.63]	−0.04 [−0.09 to 0.01]	−0.04 [−0.09 to 0.01]
African Union	1,344.83 [1,051.98–1,704.49]	48.85 [38.36–61.47]	181.92 [117.5–275.61]	6.61 [4.32–10.02]	2,487.13 [1,805.22–3,378.42]	37.74 [27.55–50.59]	337.82 [206.69–526.36]	5.13 [3.12–8]	−1.41 [−1.62 to −1.2]	−1.4 [−1.61 to −1.19]
Commonwealth	2,571.39 [2,005.27–3,324.92]	38.53 [30.04–48.83]	347.98 [220.84–534]	5.21 [3.31–7.97]	4,707.47 [3,496.4–6,209.99]	34.75 [25.87–45.81]	639.56 [385.84–982.63]	4.72 [2.84–7.25]	−0.71 [−0.84 to −0.57]	−0.69 [−0.82 to −0.56]
G20	4,220.23 [3,158.52–5,443.33]	22.93 [17.22–29.53]	575.25 [354.14–881.86]	3.13 [1.93–4.81]	6,452.64 [4,868.15–8,361.48]	25.05 [18.88–32.08]	879.52 [535.31–1,344.17]	3.41 [2.1–5.22]	0.17 [0.13 to 0.21]	0.17 [0.13 to 0.21]
OECD Countries	1,331.74 [987.23–1,744.54]	22.63 [16.77–29.62]	182.66 [107.39–278.36]	3.1 [1.83–4.73]	1,671.57 [1,294.93–2,112.29]	24.84 [19.21–31.2]	228.82 [142.76–339.5]	3.4 [2.14–5.05]	0.13 [0.03 to 0.23]	0.13 [0.03 to 0.22]
Four World Regions	6,634.5 [5,082.82–8,458.38]	25.82 [19.67–32.83]	902.85 [565.65–1,368.47]	3.51 [2.2–5.33]	10,884.58 [8,143.41–14,041.61]	27.02 [20.24–34.9]	1,482.58 [914.21–2,267.67]	3.68 [2.27–5.63]	−0.04 [−0.09 to 0.01]	−0.04 [−0.09 to 0.01]
Gulf Cooperation Council	13.48 [9.68–17.82]	16.48 [12.03–21.76]	1.84 [1.09–2.92]	2.25 [1.33–3.56]	54.65 [38.41–74.1]	16.34 [11.97–21.76]	7.45 [4.27–11.68]	2.23 [1.29–3.41]	−0.05 [−0.08 to −0.02]	−0.04 [−0.08 to −0.01]
Organization of Islamic Cooperation	1,507.49 [1,185.91–1,907.99]	33.29 [26.26–41.7]	204.37 [131.81–306.96]	4.51 [2.92–6.81]	2,825.43 [2,081.75–3,760.14]	28.28 [20.87–37.51]	384.11 [231.52–590.82]	3.84 [2.33–5.88]	−0.87 [−1.06 to −0.68]	−0.86 [−1.05 to −0.67]
Nordic Region	12.95 [9.65–16.99]	10.79 [8.1–13.99]	1.78 [1.05–2.72]	1.48 [0.88–2.26]	16.96 [12.49–22.29]	13.22 [9.58–17.37]	2.33 [1.36–3.63]	1.82 [1.05–2.84]	0.65 [0.04 to 1.27]	0.65 [0.04 to 1.26]
Health System Grouping Levels	6,639.63 [5,086.67–8,465.04]	25.82 [19.67–32.83]	903.55 [566.06–1,369.49]	3.51 [2.2–5.33]	10,888.85 [8,146.65–14,046.89]	27.02 [20.24–34.89]	1,483.17 [914.56–2,268.54]	3.68 [2.27–5.63]	−0.04 [−0.09 to 0.01]	−0.04 [−0.09 to 0.01]
Association of Southeast Asian Nations	226.02 [164.88–296.75]	10.5 [7.73–13.69]	30.97 [18.32–47.58]	1.44 [0.85–2.22]	378.21 [283.13–487.32]	10.25 [7.68–13.21]	51.65 [31.85–80.14]	1.4 [0.86–2.16]	−0.12 [−0.17 to −0.07]	−0.13 [−0.18 to −0.08]
Sahel Region	448.28 [356.96–554.88]	72.68 [58.68–89.74]	60.51 [39.29–88.9]	9.81 [6.36–14.38]	819.64 [585.64–1,136.32]	47.62 [34.54–65.29]	111.08 [67.24–170.7]	6.45 [3.97–9.96]	−2.07 [−2.39 to −1.76]	−2.06 [−2.37 to −1.74]

Further, we also analyzed the prevalence and YLDs of PID associated with chlamydia, gonococcal and other STIs. Similarly, the global prevalence and YLDs of PID did not change obviously for the chlamydia and other STIs. Interestingly, the EAPC for the ASPR (High SDI: −1.21 [−1.29 to −1.13]; High-middle SDI: −0.6 [−0.67 to −0.53]; Middle SDI: −0.12 [−0.18 to −0.05]; Low-middle SDI: −1.11 [−1.25 to −0.97]; Low SDI: −1.97 [−2.21 to −1.73]) and YLDs (High SDI: −1.21 [−1.29 to −1.13]; High-middle SDI: −0.6 [−0.67 to −0.54]; Middle SDI: −0.11 [−0.17 to −0.05]; Low-middle SDI: −1.1 [−1.24 to −0.96]; Low SDI: −1.95 [−2.19 to −1.7]) of gonococcal-associated PID all significantly decreased in all the SDI regions (Supplementary Tables S1–S3).

### PID in different countries all over the world

**Figure 1:**
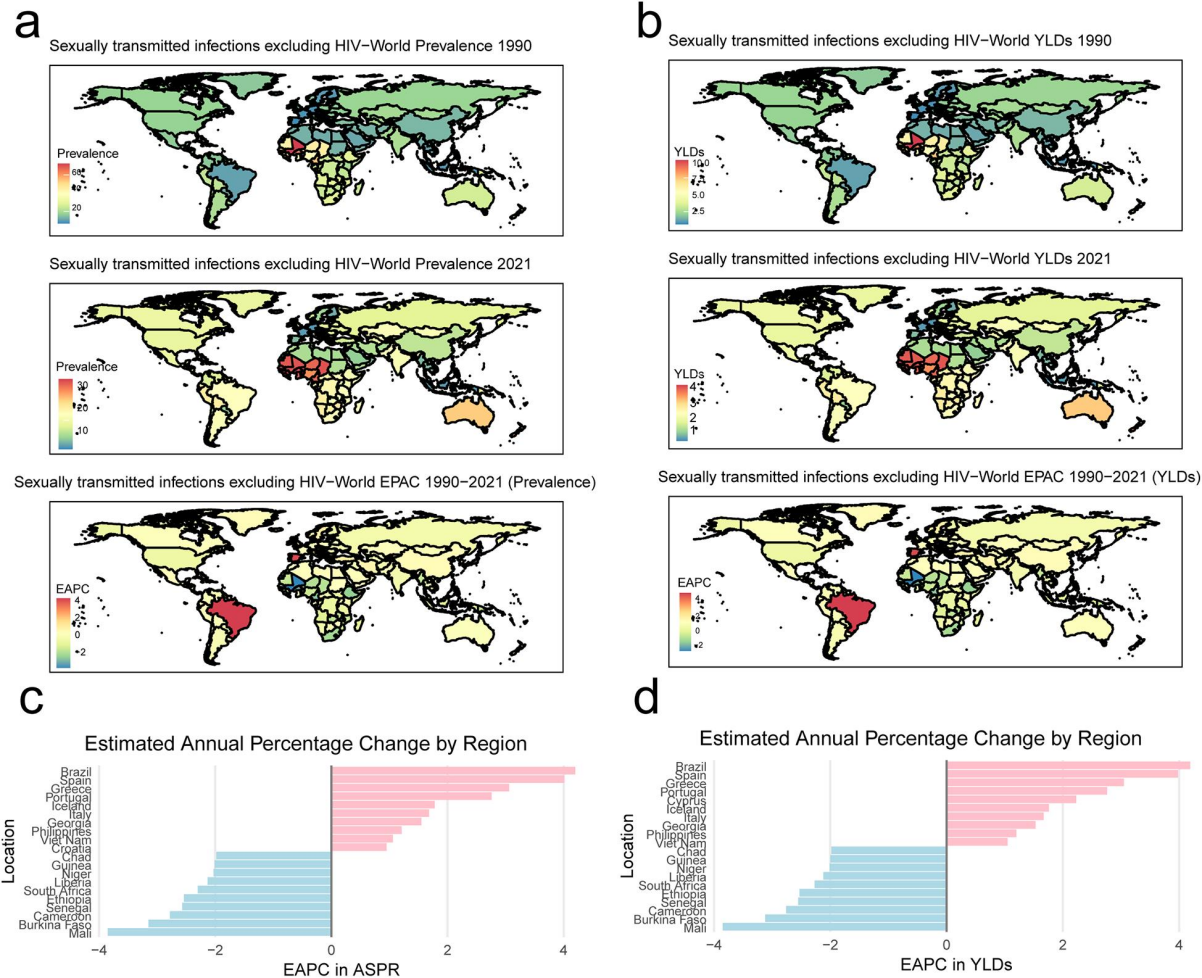
The prevalence and years lived with disability (YLDs) of sexually transmitted infection (STI) excluding HIV-associated pelvic inflammatory disease (PID) in the world in 1990 and 2021 year. **(a)** The prevalence of non-HIV PID and the estimated annual percentage changes (EAPC); **(b)** the YLDs of non-HIV PID and the estimated annual percentage changes (EAPC); **(c,d)** ten countries with the highest and lowest EAPC in age-standardized prevalence rates (ASPR) and age-standardized YLDs.

Then, the prevalence and YLDs of non-HIV PID in 204 countries were also evaluated. As shown in the world map, the number of both prevalence and YLDs of non-HIV PID increased from 1990 to 2021 (Figures 1a,b). The EAPC of ASPR and age-standardized YLDs were higher in the Brazil (4.19 [2.92, 5.47]), Spain (3.98 [3.19, 4.77]), Greece (3.05 [2.55, 3.55]), Portugal (2.76 [2.22, 3.29]), Cyprus (2.23 [0.84, 3.64]), Iceland (1.76 [0.65, 2.88]), Italy (1.67 [1.13, 2.21]), Georgia (1.53 [1.25, 1.80]) and Philippines (1.20 [0.99, 1.42]), which suggested the increased burden of non-HIV PID (Figures 1c,d). Ten countries (including Mali, Burkina Faso, Cameroon, Senegal, Ethiopia, South Africa, Liberia, Niger, Guinea, Chad and Viet Nam) effectively controlled the non-HIV PID in last 30 years (Figures 1c,d).

Then, we also analyzed the APC of non-HIV PID among the countries with the best and worst disease control by applying the Joinpoint software. The results suggested that the APC of prevalence and YLDs decreased synchronously in 1996 in the Mali, Burkina Faso, Cameroon, Senegal and Ethiopia. However, in 2009, the decline had reached a plateau. Even in 2016, a slight rise presented. As for the Brazil, since 1995, the prevalence and YLDs had increased. Although, they descended in 2015, the prevalence and YLDs were also higher than other countries (Supplementary Figure S1).

**Figure 2:**
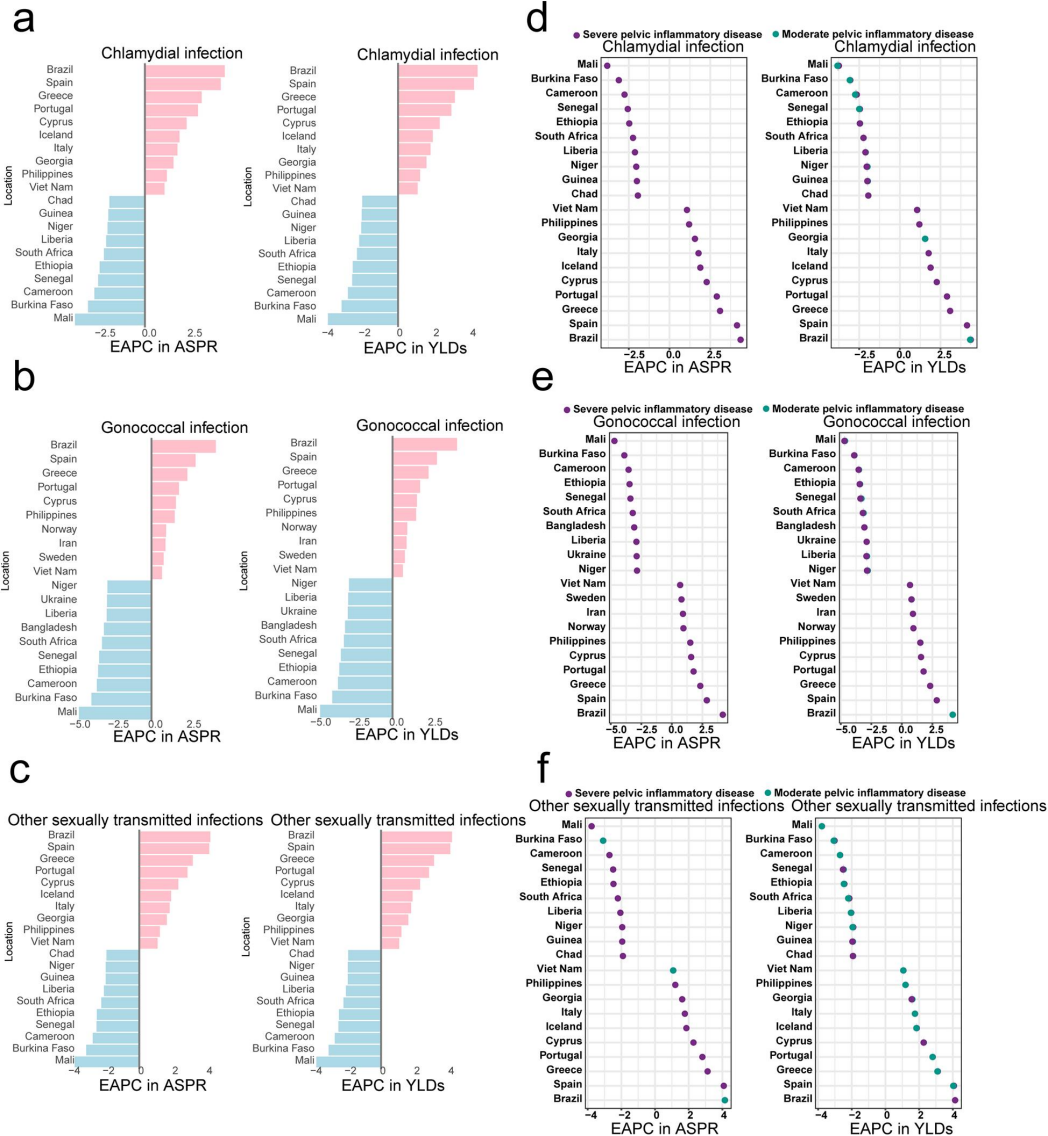
The top ten countries with the significant estimated annual percentage changes (EAPC) in age-standardized prevalence rates (ASPR) and age-standardized years lived with disability (YLDs) of pelvic inflammatory disease (PID) attributed to chlamydia, gonococcal and other sexually transmitted infection (STI). **(a–c)** Top ten EAPC in ASPR and YLDs for the chlamydia, gonococcal and other sexually transmitted infection (STI) associated PID; **(d–f)** the trends of severe and moderate PID attributed to the chlamydia, gonococcal and other STI.

Moreover, we also analyzed the tendency of prevalence and YLDs of PID correlated to the chlamydia, gonococcal and other STIs respectively. Brazil and Spain had still the highest growth rate of ASPR and YLDs. Moreover, the Mail and Burkina Faso effectively controlled the PID (Figures 2a–c). Interestingly, Norway, Iran and Sweden presented to have high EAPC in the ASPR (Norway: 0.95 [0.29, 1.62]; Iran: 0.91 [0.61, 1.21], Sweden: 0.79 [0.17, 1.42]) and YLDs (Norway: 0.95 [0.29, 1.62]; Iran: 0.91 [0.61, 1.21], Sweden: 0.80 [0.17, 1.43]) especially for the gonococcal-associated PID. Additionally, we found the EPAC of severe PID in ASPR and YLDs all increased comparing to the moderated PID correlated to the sexually transmitted infection excluding HIV from 1990 to 2021 (Figures 2d–f).

### Epidemiological analysis of PID based on the age and SDI

**Figure 3:**
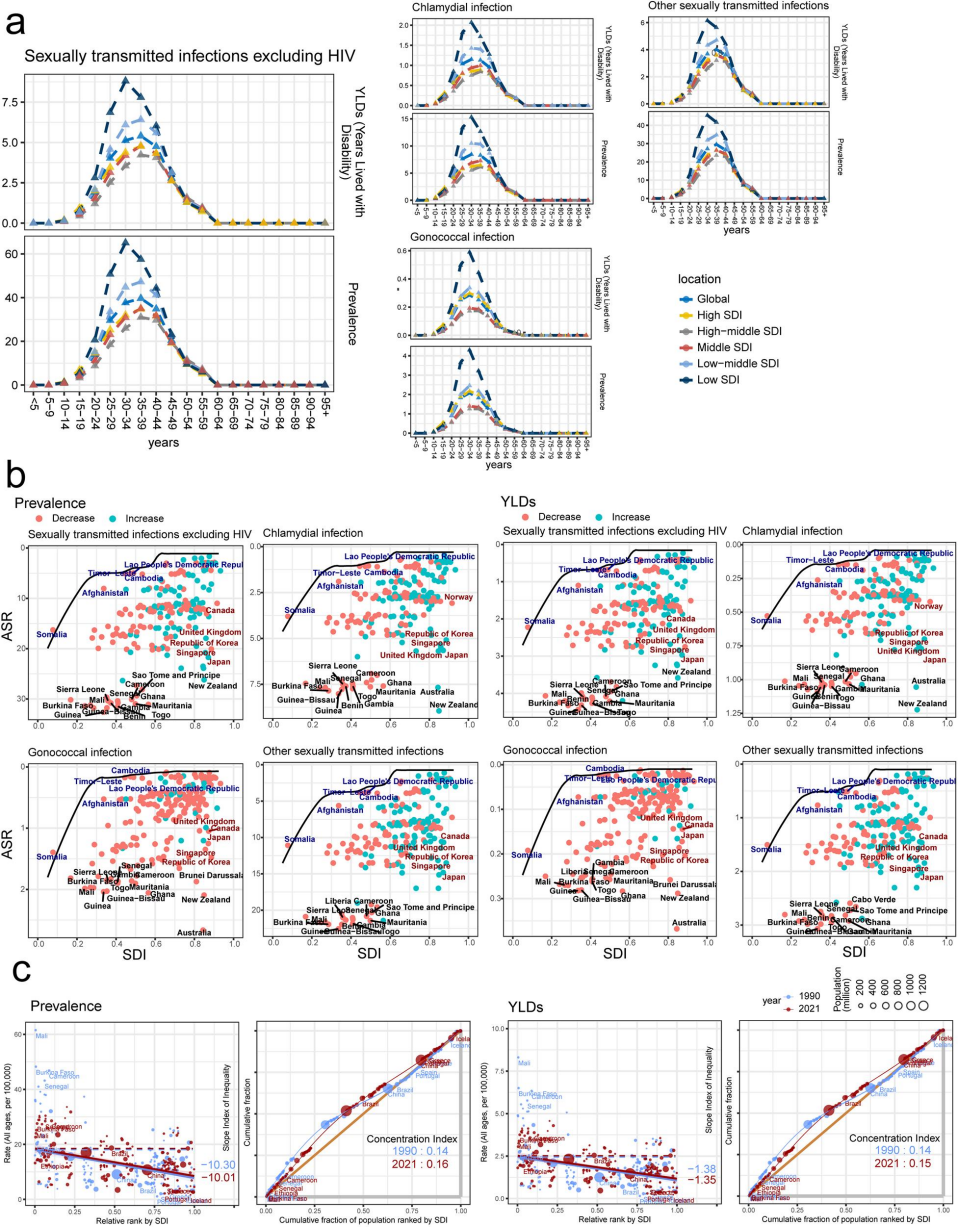
The effects of social development defined by socioDemographic Index (SDI) in the prevalence and years lived with disability (YLDs) of sexually transmitted infection (STI) excluding HIV-associated pelvic inflammatory disease (PID). **(a)** The prevalence and YLDs in different SDI regions and ages; **(b,c)** frontier analysis **(b)** and health inequality analysis **(c)** identified the association between the SDI and PID prevalence.

According to the SDI, the global regions were divided into five parts (High SDI, High-middle SDI, Middle SDI, Low-middle SDI and Low SDI). And the non-HIV PID was most commonly found in the low SDI and low-middle SDI (Figure 3a). Additionally, the non-HIV PID happened from 10 to 14 years, which rose continuously to 30–34 years and reached the peaks. Then, at 55–59 years, the prevalence and YLDs almost disappeared (Figure 3a). The variation tendency was similar in the specific PID-causing bacteria (Figure 3a). Frontier analysis identified possibility of improvement for near all the countries, especially for the high SDI, such as United Kingdom, Canada, Japan, Singapore, Korea, Brunel Darussalam, New Zealand, and Australia (Figure 3b). Health inequality analysis found the slope index of inequality for the prevalence was −10.30 in 1990. And a slight increase of slope index in 2021 hinted the more efforts were needed to control the non-HIV PID (Figure 3c). The relatively minor change was also presented in the chlamydia, gonococcal and other STIs (Supplementary Figure S2).

### Forecast of the Non-HIV PID in the last 30 years

**Figure 4:**
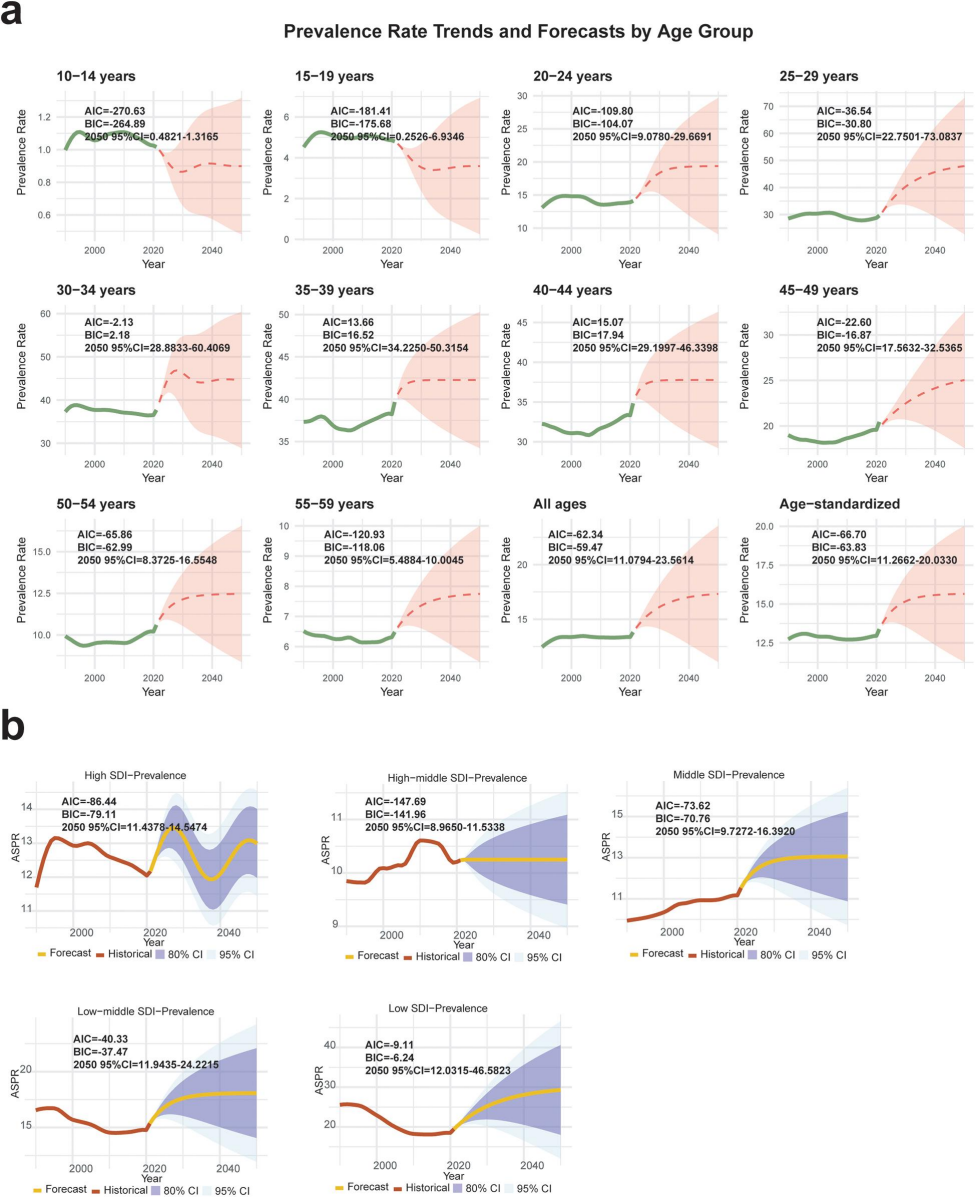
Autoregressive integrated moving average (ARIMA) model was used to forecast the prevalence of sexually transmitted infection (STI) excluding HIV-associated pelvic inflammatory disease (PID) in the different age groups **(a)** and SDI regions **(b)**.

In order to help design the strategies for the disease control, we forecasted the prevalence and YLDs in the last 30 years to 2050. As shown in the ARIMA model, we found that the overall prevalence and YLDs would rise parabolically again (Figure 4a, Supplementary Figure S3a). Although the non-HIV PID began in 10–14 years, the further significant decrease of prevalence would show among the age 10–19 in 2050 (95%CI = 0.2526–6.9346) (Figure 4a, Supplementary Figure S3a). In the other ages, both the prevalence and YLDs raised, especially for the women from 25 to 34 years (25–29 years: prevalence 95%CI = 22.7501–73.0837, YLDs 95%CI = 2.8965–6.7990; 30–34 years: prevalence 95%CI = 28.8833–60.4069, YLDs 95%CI = 4.4232–6.5140) (Figure 4a, Supplementary Figure S3a). As for the SDI regions, the prevalence and YLDs were also increased, especially in the middle SDI (ASPR 95%CI = 9.7272–16.3920, ASYLDs 95%CI = 1.3370–2.1468), low-middle SDI (ASPR 95%CI = 11.9435–24.2215, ASYLDs 95%CI = 1.6247–3.2688) and low SDI regions (ASPR 95%CI = 12.0315–46.5823, ASYLDs 95%CI = 1.6133–6.1838) (Figure 4b, Supplementary Figure S3b).

## Discussions

This study was mainly focused on the epidemiology of non-HIV PID, which might be neglected comparing to the HIV prevention. The results had exhibited the actual prevalence of non-HIV PID. based on the GBD database. We had discovered some key points. Firstly, the stable EAPC in ASPR and YLDs of non-HIV PID was shown from 1990 to 2021 globally. Secondly, some countries (especially for the Brazil, Spain and Greece) still demonstrated increased prevalence and EAPC in the non-HIV PID even in the developed regions, which needed more attentions. Thirdly, the emergence of non-HIV PID started from 10 to 14 years and reached the peaks in 30–34 years, which updated the opinion that PID was most common in aged 20–29 (3).

Till now, due to the hidden symptoms, the actual prevalence of non-HIV PID had been unclear (6, 7). In 2019, there was 9,535·71 per 100,000 person-years (8,169·73–11,054·76) for the STIs (including syphilis, chlamydia, gonorrhoea, trichomonas, and genital herpes) (15). But the rate of clinical PID diagnoses was 281/100,000 person-years (3). Although these studies could provide the bulk prevalence of PID and STIs, the detailed data for the STIs-associated PID had been negligent. In order to address these issues, the analysis was conducted comprehensively. In our data, the ASPR and age-standardized YLDs of chlamydial-associated PID were 6.22/100,000 and 0.85/100,000. As for the gonococcal-associated PID, the ASPR and age-standardized YLDs were 1.31 and 0.18 per 100,000. And the other non-HIV PID would show in 19.49/100,000 (ASPR) and 2.65/100,000 (age-standardized YLDs) in 2021. These data would provide more accurate epidemiological evidences to control the burden of diseases.

Moreover, both the absolute cases of EAPC in ASPR and YLDs increased from 1990 to 2021, which might be associated with the raised incident cases of STIs (15). Additionally, in the high-middle SDI and middle SDI, the EAPC in prevalence and YLDs increased, which suggested no positive correlation of developed level of the economy and society with non-HIV PID. So, non-HIV STIs should also not be ignored in the developed regions.

Interestingly, the EAPC in ASPR and YLDs of PID correlated to the gonococcal infection significantly decreased near in all the SDI regions. As for the countries, Mail, Burkina Faso and Cameroon were top three with the significantly decreased gonorrhoeae-associated PID. In 1996, a Sweden study had identified a steady decline in gonorrhoeae-associated PID by following 25 years in approximately 2,500 patients (16). Moreover, in the United Kingdom, United States, and Australia, the prevalence of gonorrhoeae-associated PID was also decreased these years (17). The decline might acknowledge the aggressive public health measures, including publicity efforts, actively screens for gonorrhoeae, and higher adoption rates of safe sex practices (13). Another interesting point was that three countries (Brazil, Spain and Greece), showed the highest rate of elevation in the EAPC (ASPR and YLDs) for the non-HIV PID (including the chlamydia, gonococcal and other sexually transmitted infections). These results indicated the task of prevention of PID was still arduous and required a long-term effort especially for the three countries. More powerful promotion, widespread screening for pathogens and safe sex practices should also be carried out.

Above all, non-HIV PID was not a disease specific to underdeveloped regions. In the high SDI regions, the STIs were still troublesome health issues (15). In the frontier analysis and health inequality analysis, we also identified a greater room for improvements in the developed countries, such as United Kingdom, Canada, Japan, Singapore, Korea, Brunel Darussalam, New Zealand, and Australia. So, we proposed that in these countries more publicity efforts, actively screens and safe sex practices should be adopted especially for the women in 30–34 years. Further studies should dissect the insufficient policies in these countries to improve public health policies in the non-HIV PID.

### Limitations

This study had drawn the landscape of non-HIV PID epidemiologically. Some limitations should be also noticed: 1) there were no detailed descriptions of diagnostic criteria for the PID in the GBD database. So, the diagnostic variability and underreporting could not be evaluated. 2) No explicit definitions for the moderate and severe PID would limit the further analyses.

## Conclusions

The prevalence of non-HIV PID was still persistently increased globally, even in the developed countries. Considering the potential growth trend of non-HIV PID in the next 30 years, more medical attentions, publicity efforts, actively screens for non-HIV STIs, and safe sex practices should be implemented especially for the 30–34 year's females.

## Data Availability

The original contributions presented in the study are included in the article/Supplementary Material, further inquiries can be directed to the corresponding author.

## Supplementary material

Supplementary Figure S1 The trends of prevalence and years lived with disability (YLDs) of sexually transmitted infection (STI) excluding HIV-associated Pelvic inflammatory disease (PID) among the countries with greatest changes.

Supplementary Figure S2

Health inequality analysis for the prevalence and years lived with disability (YLDs) of Pelvic inflammatory disease (PID) attributed to the chlamydia, gonococcal and other sexually transmitted infection (STI).

Supplementary Figure S3

Autoregressive Integrated Moving Average (ARIMA) model was used to forecast the years lived with disability (YLDs) of sexually transmitted infection (STI) excluding HIV-associated Pelvic inflammatory disease (PID) in the different age groups (a) and SDI regions (b).
