# Supplementary material for: Global burden and trends of pelvic inflammatory disease associated with sexually transmitted infection excluding HIV from 1990 to 2021
Source: Front Glob Womens Health. 2025 Dec 16;6:1658086. doi: 10.3389/fgwh.2025.1658086 (PMC12747987; doi:10.3389/fgwh.2025.1658086)
Supplement: Supplementary Figure S1 — The trends of prevalence and years lived with disability (YLDs) of sexually transmitted infection (STI) excluding HIV-associated Pelvic inflammatory disease (PID) among the countries with greatest changes. [file Table1.docx]

**Figure S1**


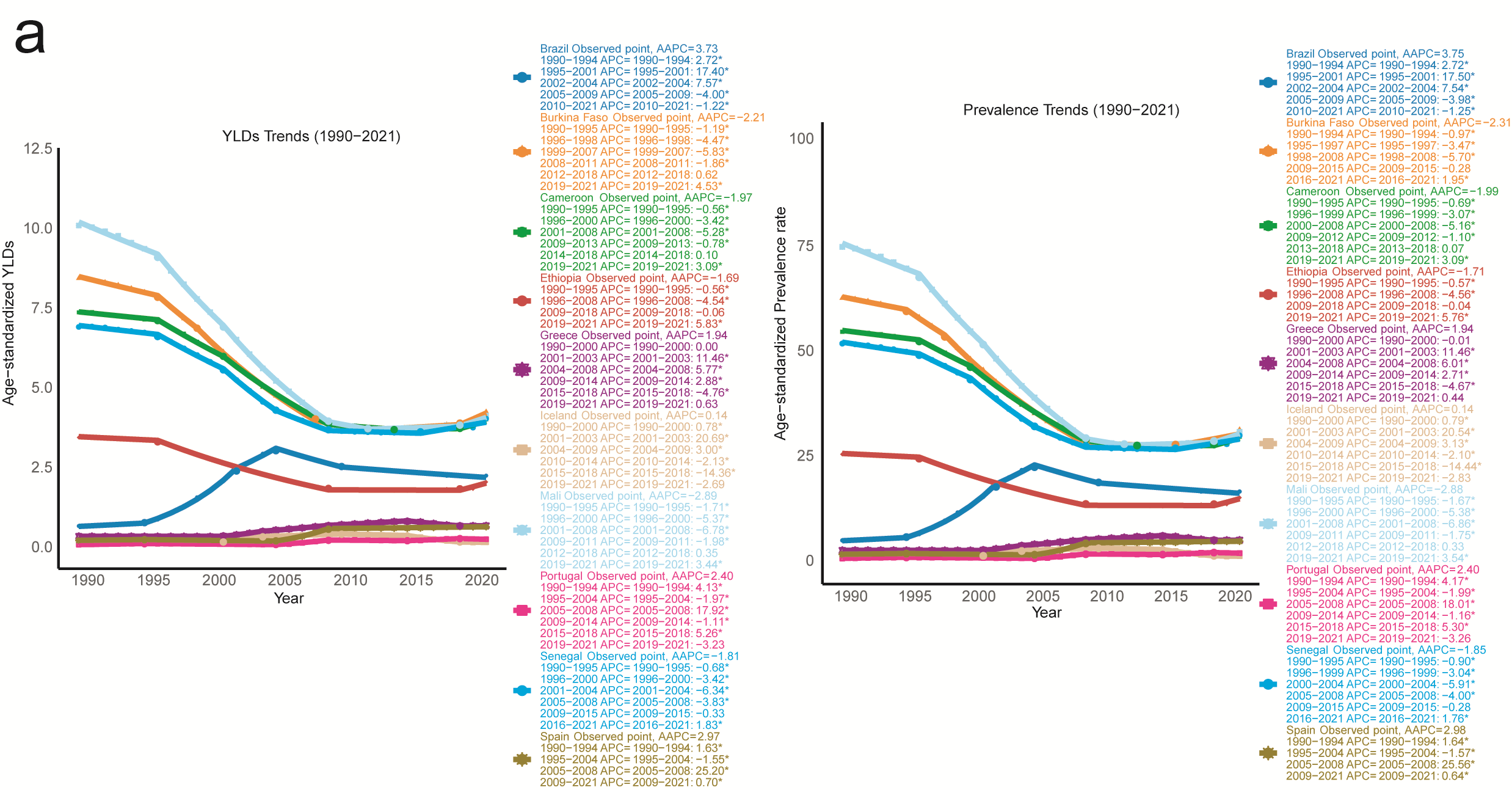


**Figure S2**


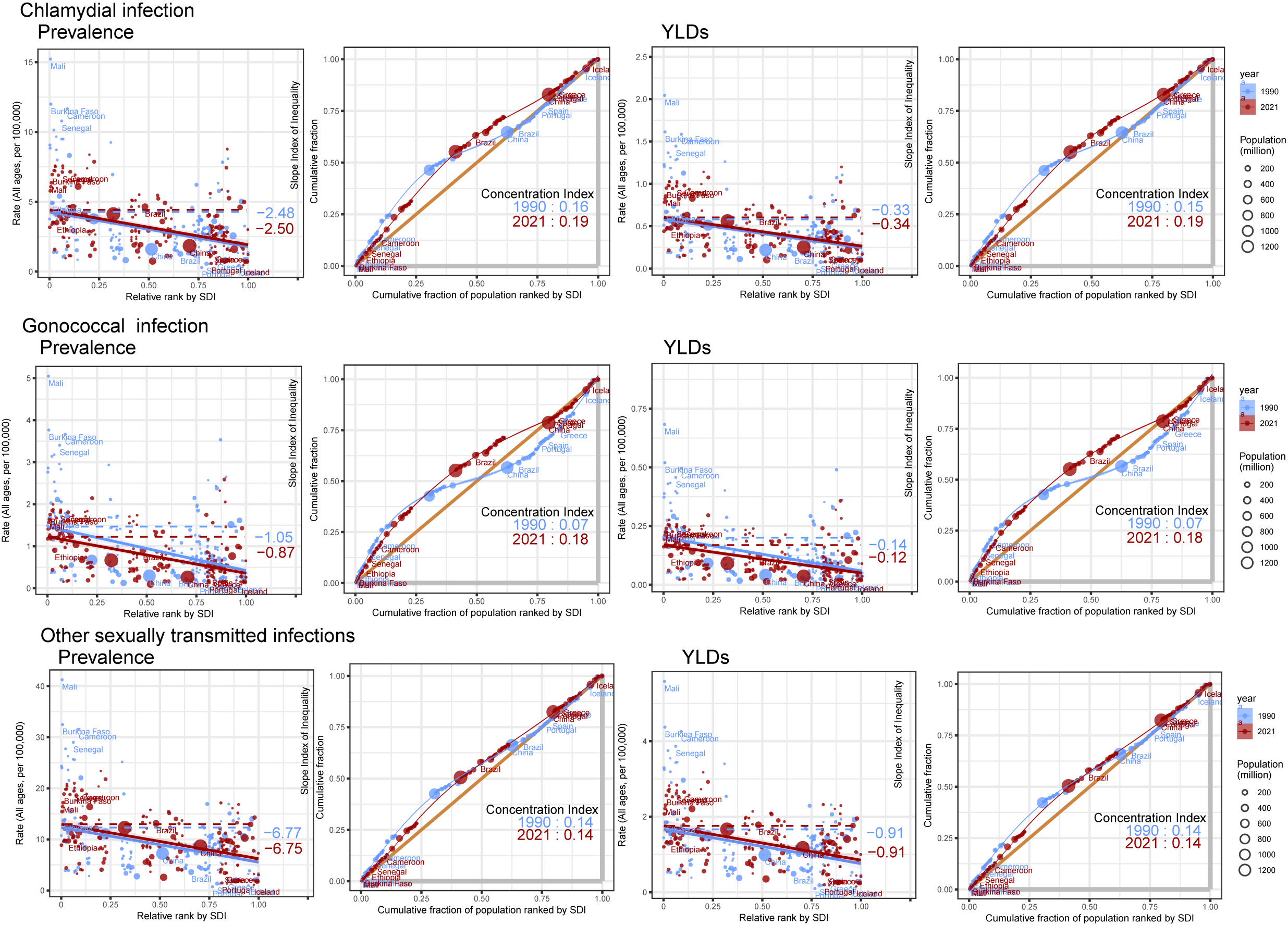


**Figure S3**


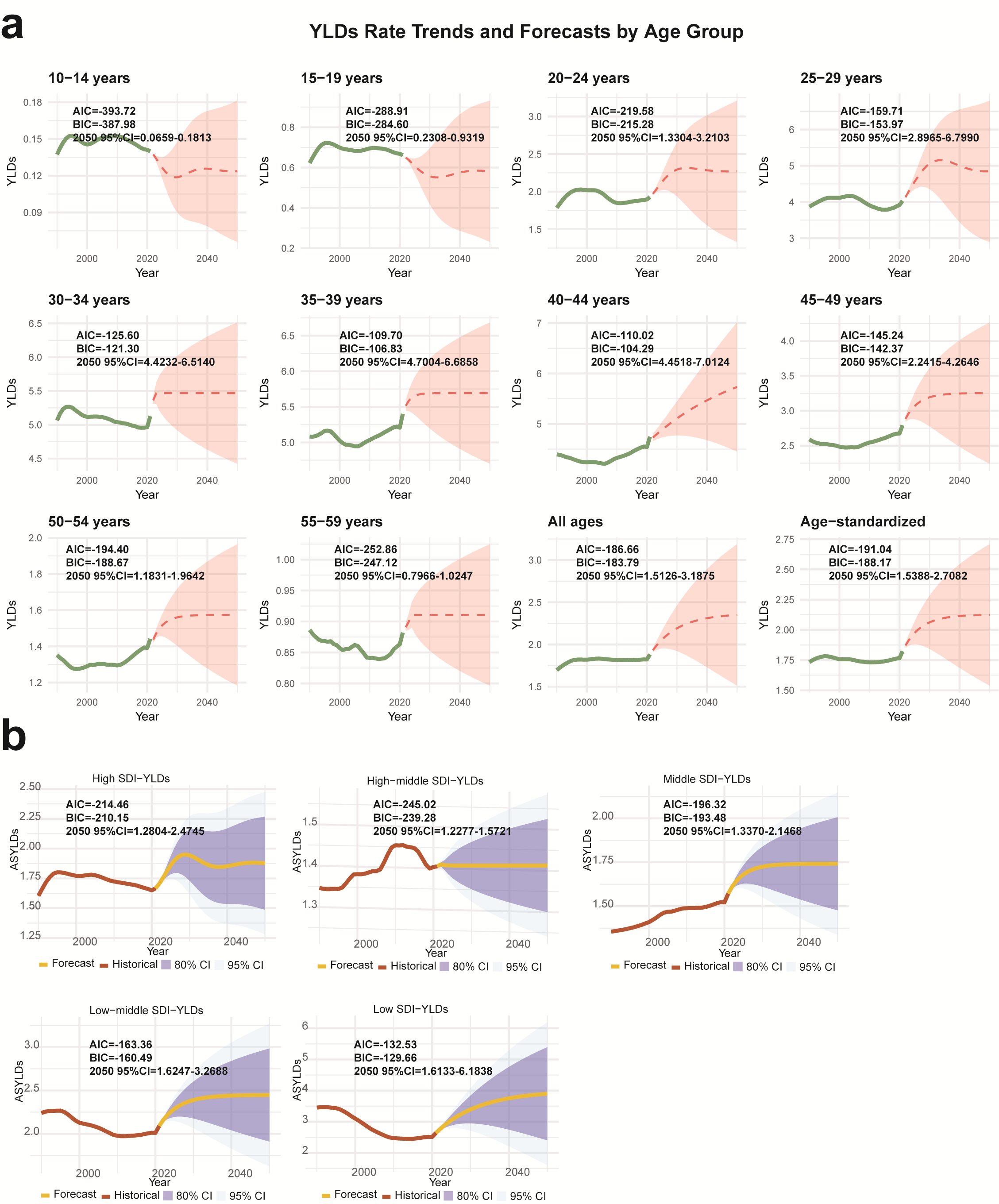


|  |  | 1990 |  |  |  |  | 2021 |  |  |  |  | | 1990-2021 |
| --- | --- | --- | --- | --- | --- | --- | --- | --- | --- | --- | --- | --- | --- |
|  | Prevalent cases No. *102 (95% UI) | ASPR per 100,000 No. (95% UI) | YLD cases No. *102 (95% UI) | Age-standardized YLD rate per 100,000 No. (95% UI) |  | Prevalent cases No. *102 (95% UI) | ASPR per 100,000 No.(95% UI) | YLD cases No. *102 (95% UI) | Age-standardized YLD rate per 100,000 No.(95% UI) |  | EAPC of ASPR No. (95% CI) | | EAPC of Age-standardized YLD rate No. (95% CI) |
| Global | 1518.29 [1151.73-2032.15] | 5.8 [4.43-7.7] | 207.3 [126-320.25] | 0.79 [0.49-1.21] |  | 2489.44 [1853.4-3368.72] | 6.22 [4.61-8.4] | 340.21 [204.81-522.49] | 0.85 [0.51-1.31] |  | 0.04 [-0.02 to 0.1] | | 0.04 [-0.02 to 0.1] |
| Socio-demographic index |  |  |  |  |  |  |  |  |  |  | |  |  |
| High SDI | 247.34 [182.78-334.96] | 5.3 [3.9-7.13] | 33.95 [20.09-53.5] | 0.73 [0.43-1.14] |  | 276.49 [212.1-360.71] | 5.36 [4.09-6.99] | 37.94 [23.22-57.13] | 0.73 [0.45-1.12] |  | | -0.13 [-0.2 to -0.06] | -0.14 [-0.21 to -0.07] |
| High-middle SDI | 238.01 [176.29-323.07] | 4.28 [3.17-5.74] | 32.65 [19.94-51.08] | 0.59 [0.36-0.92] |  | 311.66 [234.41-413.05] | 4.55 [3.44-6.11] | 42.77 [25.58-68.23] | 0.62 [0.38-0.98] |  | | 0.29 [0.24 to 0.34] | 0.29 [0.23 to 0.34] |
| Middle SDI | 362.07 [269.09-490.21] | 4.31 [3.24-5.82] | 49.57 [29.51-77.24] | 0.59 [0.36-0.91] |  | 684.71 [515.35-910.88] | 5.26 [3.95-7.04] | 93.68 [57.53-141.37] | 0.72 [0.44-1.1] |  | | 0.56 [0.51 to 0.61] | 0.56 [0.51 to 0.61] |
| Low-middle SDI | 409.8 [312.56-542.25] | 7.88 [5.98-10.26] | 55.72 [33.75-85.83] | 1.07 [0.66-1.64] |  | 737.36 [539.82-1013.63] | 7.35 [5.41-10.07] | 100.65 [59.48-157.78] | 1 [0.6-1.57] |  | | -0.49 [-0.61 to -0.38] | -0.48 [-0.59 to -0.37] |
| Low SDI | 259.77 [201.04-337.32] | 12.04 [9.42-15.49] | 35.22 [21.86-53.44] | 1.63 [1.01-2.45] |  | 477.68 [343.92-675.21] | 9.2 [6.67-12.67] | 64.97 [37.36-103.33] | 1.25 [0.72-1.98] |  | | -1.38 [-1.59 to -1.16] | -1.36 [-1.56 to -1.15] |
| Region |  |  |  |  |  |  |  |  |  |  | |  |  |
| Southeast Asia, East Asia, and Oceania | 246.23 [182.09-335.36] | 2.91 [2.16-3.9] | 33.88 [19.82-53.16] | 0.4 [0.24-0.63] |  | 354.47 [266.39-465.62] | 3.03 [2.27-4.06] | 48.75 [29.33-76.19] | 0.42 [0.26-0.65] |  | | 0.2 [0.07 to 0.32] | 0.19 [0.07 to 0.31] |
| North Africa and Middle East | 58.78 [43.3-80] | 4.08 [2.97-5.56] | 8.09 [4.75-12.88] | 0.56 [0.33-0.89] |  | 133.24 [95.73-182.22] | 4.11 [2.98-5.6] | 18.34 [10.81-29.44] | 0.57 [0.34-0.9] |  | | 0.24 [0.14 to 0.34] | 0.23 [0.14 to 0.33] |
| Central Europe, Eastern Europe, and Central Asia | 138.12 [103.08-185.68] | 6.32 [4.73-8.49] | 18.9 [11.31-29.61] | 0.86 [0.52-1.35] |  | 137.6 [103.09-184.87] | 6.37 [4.78-8.57] | 18.87 [11.43-30.07] | 0.87 [0.53-1.39] |  | | 0 [-0.09 to 0.09] | 0.01 [-0.08 to 0.09] |
| Latin America and Caribbean | 89.57 [67.1-120.01] | 4.7 [3.56-6.23] | 12.28 [7.28-19.01] | 0.64 [0.39-0.98] |  | 234.33 [179.46-308.22] | 7.21 [5.55-9.52] | 32.02 [19.61-49.56] | 0.99 [0.61-1.53] |  | | 1.55 [1.06 to 2.05] | 1.54 [1.05 to 2.03] |
| High-income | 253.29 [187.49-342.7] | 5.29 [3.91-7.15] | 34.75 [20.48-54.4] | 0.73 [0.43-1.13] |  | 286.51 [218.78-373.34] | 5.68 [4.34-7.39] | 39.29 [24.07-59.39] | 0.78 [0.48-1.2] |  | | 0.1 [0 to 0.2] | 0.09 [-0.01 to 0.19] |
| South Asia | 421.82 [321.46-558.27] | 8.63 [6.56-11.32] | 57.3 [35.04-89.31] | 1.17 [0.73-1.81] |  | 774.67 [566.63-1058.41] | 7.9 [5.79-10.77] | 105.5 [62.4-166.11] | 1.08 [0.64-1.69] |  | | -0.56 [-0.68 to -0.44] | -0.55 [-0.67 to -0.43] |
| Sub-Saharan Africa | 310.48 [233.88-407.17] | 14.23 [10.77-18.46] | 42.09 [25.49-64.62] | 1.93 [1.17-2.94] |  | 568.62 [394.08-819.19] | 10.48 [7.37-14.84] | 77.44 [44.38-125.42] | 1.43 [0.83-2.28] |  | | -1.62 [-1.84 to -1.4] | -1.6 [-1.82 to -1.39] |
| WHO region | 1512.7 [1147.3-2024.58] | 5.81 [4.44-7.71] | 206.53 [125.52-319.07] | 0.79 [0.49-1.21] |  | 2483.08 [1848.79-3359.84] | 6.23 [4.62-8.41] | 339.33 [204.23-521.27] | 0.85 [0.51-1.31] |  | | 0.03 [-0.02 to 0.09] | 0.04 [-0.02 to 0.1] |
| European Union | 68.52 [50.8-93.24] | 3.15 [2.32-4.31] | 9.42 [5.6-14.96] | 0.43 [0.26-0.69] |  | 77.67 [59.83-100.04] | 3.79 [2.93-4.89] | 10.66 [6.53-16.24] | 0.52 [0.32-0.81] |  | | 0.78 [0.57 to 1] | 0.78 [0.56 to 0.99] |
| World Bank Regions | 1516.43 [1150.28-2029.73] | 5.8 [4.44-7.7] | 207.05 [125.84-319.87] | 0.79 [0.49-1.21] |  | 2487.25 [1851.78-3365.69] | 6.22 [4.61-8.4] | 339.91 [204.63-522.02] | 0.85 [0.51-1.31] |  | | 0.04 [-0.02 to 0.1] | 0.04 [-0.02 to 0.1] |
| African Union | 333.56 [251.27-437.76] | 11.82 [8.98-15.43] | 45.27 [27.32-69.6] | 1.6 [0.97-2.44] |  | 618.1 [432.55-885.38] | 9.21 [6.51-12.95] | 84.26 [48.33-136.41] | 1.26 [0.72-2.01] |  | | -1.39 [-1.6 to -1.18] | -1.37 [-1.58 to -1.17] |
| Commonwealth | 629.96 [475.44-843.42] | 9.22 [6.97-12.19] | 85.6 [51.65-132.22] | 1.25 [0.77-1.92] |  | 1152.29 [840.28-1585.03] | 8.43 [6.19-11.56] | 157.06 [92.64-246.64] | 1.15 [0.68-1.79] |  | | -0.68 [-0.83 to -0.53] | -0.67 [-0.82 to -0.52] |
| G20 | 931.32 [695.53-1255.81] | 4.99 [3.76-6.69] | 127.34 [77.32-198.62] | 0.68 [0.42-1.05] |  | 1415.19 [1074.22-1897.05] | 5.6 [4.21-7.48] | 193.44 [118.02-292.77] | 0.76 [0.46-1.16] |  | | 0.26 [0.22 to 0.31] | 0.26 [0.21 to 0.3] |
| OECD Countries | 305.4 [225.25-415.3] | 5.22 [3.84-7.07] | 41.93 [24.81-66.06] | 0.72 [0.42-1.13] |  | 369.91 [284.82-483.24] | 5.62 [4.28-7.35] | 50.72 [31.15-77.22] | 0.77 [0.48-1.18] |  | | 0.17 [0.08 to 0.25] | 0.16 [0.08 to 0.24] |
| Four World Regions | 1515.79 [1149.8-2028.94] | 5.8 [4.44-7.7] | 206.96 [125.78-319.71] | 0.79 [0.49-1.21] |  | 2486.92 [1851.56-3365.22] | 6.22 [4.61-8.4] | 339.86 [204.59-521.99] | 0.85 [0.51-1.31] |  | | 0.04 [-0.02 to 0.1] | 0.04 [-0.02 to 0.1] |
| Gulf Cooperation Council | 3.33 [2.4-4.56] | 3.94 [2.84-5.38] | 0.46 [0.28-0.73] | 0.54 [0.33-0.86] |  | 12.9 [8.99-17.87] | 3.92 [2.82-5.36] | 1.77 [1.01-2.86] | 0.54 [0.32-0.85] |  | | -0.04 [-0.07 to -0.01] | -0.04 [-0.07 to -0.01] |
| Organization of Islamic Cooperation | 369.72 [282.66-488.97] | 7.94 [6.11-10.38] | 50.33 [30.73-78.74] | 1.08 [0.67-1.65] |  | 690.91 [503.5-966.35] | 6.86 [5.01-9.51] | 94.37 [55.81-151.25] | 0.94 [0.56-1.5] |  | | -0.82 [-1.01 to -0.63] | -0.81 [-0.99 to -0.62] |
| Nordic Region | 3.09 [2.29-4.2] | 2.62 [1.94-3.56] | 0.42 [0.25-0.67] | 0.36 [0.21-0.56] |  | 3.6 [2.66-4.91] | 2.87 [2.1-3.95] | 0.5 [0.29-0.79] | 0.39 [0.23-0.63] |  | | 0.45 [-0.18 to 1.08] | 0.44 [-0.18 to 1.06] |
| Health System Grouping Levels | 1516.99 [1150.72-2030.46] | 5.8 [4.43-7.7] | 207.12 [125.89-319.97] | 0.79 [0.49-1.21] |  | 2487.9 [1852.27-3366.61] | 6.22 [4.61-8.4] | 340 [204.69-522.18] | 0.85 [0.51-1.31] |  | | 0.04 [-0.02 to 0.1] | 0.04 [-0.02 to 0.1] |
| Association of Southeast Asian Nations | 49.48 [36.32-67.66] | 2.24 [1.66-3.01] | 6.81 [3.95-10.64] | 0.31 [0.18-0.48] |  | 81.46 [60.98-108.66] | 2.22 [1.67-2.98] | 11.22 [6.77-17.32] | 0.31 [0.18-0.47] |  | | -0.05 [-0.11 to 0] | -0.06 [-0.11 to 0] |
| Sahel Region | 112.28 [84.78-148.54] | 17.8 [13.42-22.99] | 15.19 [9.45-23.11] | 2.41 [1.5-3.62] |  | 207.71 [143.51-300] | 11.78 [8.09-16.85] | 28.27 [15.82-46.63] | 1.6 [0.9-2.61] |  | | -2.05 [-2.36 to -1.74] | -2.02 [-2.33 to -1.71] |

Table S1. The prevalence and years lived with disability (YLDs) of pelvic inflammatory disease correlated to Chlamydial infection in the GBD super regions. ASPR: age-standardized prevalence rates; EAPC: estimated annual percentage change

|  |  | 1990 |  |  |  |  | 2021 |  |  |  |  | | 1990-2021 |
| --- | --- | --- | --- | --- | --- | --- | --- | --- | --- | --- | --- | --- | --- |
|  | Prevalent cases No. *102 (95% UI) | ASPR per 100,000 No. (95% UI) | YLD cases No. *102 (95% UI) | Age-standardized YLD rate per 100,000 No. (95% UI) |  | Prevalent cases No. *102 (95% UI) | ASPR per 100,000 No.(95% UI) | YLD cases No. *102 (95% UI) | Age-standardized YLD rate per 100,000 No.(95% UI) |  | EAPC of ASPR No. (95% CI) | | EAPC of Age-standardized YLD rate No. (95% CI) |
| Global | 404.45 [293.75-530.62] | 1.53 [1.12-2.01] | 55.57 [32.63-87.74] | 0.21 [0.12-0.33] |  | 521.26 [376.96-708.87] | 1.31 [0.94-1.78] | 71.75 [40.97-115.9] | 0.18 [0.1-0.29] |  | -0.8 [-0.91 to -0.68] | | -0.79 [-0.9 to -0.68] |
| Socio-demographic index |  |  |  |  |  |  |  |  |  |  | |  |  |
| High SDI | 97.42 [69.4-133] | 2.07 [1.48-2.84] | 13.43 [7.64-21.55] | 0.29 [0.16-0.45] |  | 76.81 [55.61-102.05] | 1.51 [1.11-2.02] | 10.58 [6.13-16.82] | 0.21 [0.12-0.33] |  | | -1.21 [-1.29 to -1.13] | -1.21 [-1.29 to -1.13] |
| High-middle SDI | 56.54 [40.99-77.36] | 1.01 [0.73-1.37] | 7.79 [4.48-12.57] | 0.14 [0.08-0.22] |  | 58.29 [42.57-78.9] | 0.88 [0.64-1.19] | 8.03 [4.63-13.09] | 0.12 [0.07-0.19] |  | | -0.6 [-0.67 to -0.53] | -0.6 [-0.67 to -0.54] |
| Middle SDI | 76.2 [54.87-101.93] | 0.89 [0.65-1.2] | 10.48 [6.13-16.71] | 0.12 [0.07-0.2] |  | 117.74 [85.18-158.89] | 0.91 [0.66-1.23] | 16.22 [9.28-26.01] | 0.13 [0.07-0.2] |  | | -0.12 [-0.18 to -0.05] | -0.11 [-0.17 to -0.05] |
| Low-middle SDI | 97.52 [72.43-128.24] | 1.86 [1.4-2.44] | 13.41 [7.81-20.45] | 0.26 [0.15-0.4] |  | 148.48 [106.19-206.47] | 1.47 [1.05-2.03] | 20.44 [11.43-33.62] | 0.2 [0.11-0.33] |  | | -1.11 [-1.25 to -0.97] | -1.1 [-1.24 to -0.96] |
| Low SDI | 76.44 [57.54-101.21] | 3.52 [2.7-4.65] | 10.42 [6.36-16.13] | 0.48 [0.29-0.75] |  | 119.6 [82.44-166.85] | 2.28 [1.6-3.18] | 16.44 [9.35-26.7] | 0.31 [0.18-0.51] |  | | -1.97 [-2.21 to -1.73] | -1.95 [-2.19 to -1.7] |
| Region |  |  |  |  |  |  |  |  |  |  | |  |  |
| Southeast Asia, East Asia, and Oceania | 44.47 [32.15-60.4] | 0.52 [0.37-0.71] | 6.13 [3.49-9.94] | 0.07 [0.04-0.12] |  | 50.04 [37.3-67.55] | 0.44 [0.33-0.59] | 6.9 [4-11.25] | 0.06 [0.03-0.1] |  | | -0.46 [-0.63 to -0.3] | -0.46 [-0.63 to -0.3] |
| North Africa and Middle East | 14.03 [9.92-19.18] | 0.96 [0.69-1.31] | 1.93 [1.1-3.17] | 0.13 [0.08-0.22] |  | 30.63 [21.32-41.76] | 0.94 [0.66-1.28] | 4.22 [2.43-7.05] | 0.13 [0.08-0.21] |  | | 0.16 [0.06 to 0.26] | 0.16 [0.06 to 0.26] |
| Central Europe, Eastern Europe, and Central Asia | 33.29 [23.99-45.68] | 1.53 [1.1-2.08] | 4.59 [2.66-7.44] | 0.21 [0.12-0.34] |  | 25.06 [18.06-34.24] | 1.18 [0.84-1.61] | 3.45 [2-5.64] | 0.16 [0.09-0.26] |  | | -1.33 [-1.49 to -1.17] | -1.33 [-1.49 to -1.17] |
| Latin America and Caribbean | 23.59 [17.31-31.7] | 1.22 [0.89-1.64] | 3.25 [1.86-5.15] | 0.17 [0.1-0.27] |  | 47.8 [35.12-64.21] | 1.47 [1.08-1.99] | 6.58 [3.8-10.53] | 0.2 [0.12-0.32] |  | | 0.76 [0.39 to 1.13] | 0.75 [0.39 to 1.12] |
| High-income | 104.93 [74.64-143.11] | 2.18 [1.56-2.99] | 14.46 [8.21-23.04] | 0.3 [0.17-0.48] |  | 85.77 [62.17-113.64] | 1.73 [1.27-2.32] | 11.81 [6.86-18.73] | 0.24 [0.14-0.37] |  | | -0.9 [-0.97 to -0.84] | -0.91 [-0.97 to -0.84] |
| South Asia | 88.44 [65.68-116.21] | 1.8 [1.35-2.36] | 12.18 [7.14-18.78] | 0.25 [0.15-0.38] |  | 134.71 [95.69-186.15] | 1.37 [0.97-1.87] | 18.56 [10.54-30.98] | 0.19 [0.11-0.31] |  | | -1.28 [-1.41 to -1.14] | -1.27 [-1.4 to -1.14] |
| Sub-Saharan Africa | 95.7 [69.95-128.23] | 4.37 [3.28-5.81] | 13.03 [7.85-19.96] | 0.6 [0.36-0.93] |  | 147.24 [100.2-209] | 2.7 [1.87-3.82] | 20.22 [11.5-33.03] | 0.37 [0.21-0.6] |  | | -2.23 [-2.47 to -1.99] | -2.2 [-2.44 to -1.96] |
| WHO region | 403.18 [292.87-528.94] | 1.53 [1.13-2.01] | 55.4 [32.53-87.45] | 0.21 [0.12-0.33] |  | 520.03 [376.01-707.21] | 1.31 [0.94-1.78] | 71.58 [40.87-115.63] | 0.18 [0.1-0.29] |  | | -0.8 [-0.92 to -0.69] | -0.79 [-0.91 to -0.68] |
| European Union | 17.43 [12.44-24.05] | 0.8 [0.58-1.12] | 2.4 [1.38-3.92] | 0.11 [0.06-0.18] |  | 13.99 [10.41-18.6] | 0.7 [0.51-0.94] | 1.93 [1.12-3.06] | 0.1 [0.06-0.15] |  | | -0.35 [-0.52 to -0.19] | -0.35 [-0.52 to -0.19] |
| World Bank Regions | 404.01 [293.44-530.04] | 1.53 [1.12-2.01] | 55.51 [32.59-87.64] | 0.21 [0.12-0.33] |  | 520.8 [376.63-708.27] | 1.31 [0.94-1.78] | 71.69 [40.93-115.8] | 0.18 [0.1-0.29] |  | | -0.8 [-0.91 to -0.68] | -0.79 [-0.9 to -0.68] |
| African Union | 101.37 [74.4-136.14] | 3.58 [2.68-4.74] | 13.81 [8.28-21.09] | 0.49 [0.3-0.76] |  | 158.98 [108.94-224.72] | 2.35 [1.64-3.32] | 21.84 [12.39-35.74] | 0.32 [0.18-0.53] |  | | -1.98 [-2.21 to -1.75] | -1.95 [-2.18 to -1.72] |
| Commonwealth | 149.91 [110.02-200.01] | 2.18 [1.63-2.89] | 20.59 [12.16-31.43] | 0.3 [0.18-0.47] |  | 225.5 [162.09-312.4] | 1.64 [1.17-2.26] | 31.05 [17.46-50.83] | 0.23 [0.13-0.37] |  | | -1.41 [-1.59 to -1.24] | -1.4 [-1.58 to -1.23] |
| G20 | 232.97 [168.43-313.79] | 1.24 [0.9-1.67] | 32.08 [18.41-51.16] | 0.17 [0.1-0.27] |  | 266.77 [196.23-360.03] | 1.07 [0.78-1.45] | 36.75 [20.94-59.25] | 0.15 [0.08-0.24] |  | | -0.65 [-0.69 to -0.61] | -0.65 [-0.69 to -0.61] |
| OECD Countries | 113.62 [81.24-155.06] | 1.93 [1.39-2.64] | 15.66 [8.88-24.99] | 0.27 [0.15-0.42] |  | 95.3 [69.42-126.08] | 1.47 [1.08-1.98] | 13.12 [7.63-20.84] | 0.2 [0.12-0.32] |  | | -0.97 [-1.02 to -0.93] | -0.98 [-1.03 to -0.93] |
| Four World Regions | 403.79 [293.29-529.73] | 1.53 [1.12-2.01] | 55.48 [32.58-87.59] | 0.21 [0.12-0.33] |  | 520.69 [376.54-708.11] | 1.31 [0.94-1.78] | 71.67 [40.92-115.77] | 0.18 [0.1-0.29] |  | | -0.8 [-0.91 to -0.68] | -0.79 [-0.9 to -0.68] |
| Gulf Cooperation Council | 0.79 [0.55-1.09] | 0.91 [0.64-1.23] | 0.11 [0.06-0.18] | 0.13 [0.07-0.21] |  | 2.92 [1.92-4.11] | 0.87 [0.6-1.22] | 0.4 [0.22-0.69] | 0.12 [0.07-0.2] |  | | -0.16 [-0.19 to -0.14] | -0.16 [-0.19 to -0.14] |
| Organization of Islamic Cooperation | 102.39 [76-135.13] | 2.18 [1.64-2.86] | 14.03 [8.13-21.39] | 0.3 [0.17-0.46] |  | 151.48 [105.97-211.23] | 1.5 [1.06-2.07] | 20.82 [11.91-33.88] | 0.21 [0.12-0.34] |  | | -1.67 [-1.95 to -1.39] | -1.65 [-1.93 to -1.37] |
| Nordic Region | 1.1 [0.79-1.49] | 0.93 [0.67-1.26] | 0.15 [0.09-0.25] | 0.13 [0.07-0.21] |  | 1.14 [0.8-1.56] | 0.91 [0.64-1.26] | 0.16 [0.09-0.25] | 0.13 [0.07-0.2] |  | | -0.24 [-0.87 to 0.4] | -0.24 [-0.87 to 0.4] |
| Health System Grouping Levels | 404.12 [293.52-530.19] | 1.53 [1.12-2.01] | 55.53 [32.6-87.66] | 0.21 [0.12-0.33] |  | 520.92 [376.72-708.43] | 1.31 [0.94-1.78] | 71.71 [40.94-115.82] | 0.18 [0.1-0.29] |  | | -0.8 [-0.91 to -0.68] | -0.79 [-0.9 to -0.68] |
| Association of Southeast Asian Nations | 8.35 [5.96-11.48] | 0.37 [0.27-0.5] | 1.15 [0.66-1.83] | 0.05 [0.03-0.08] |  | 11.79 [8.56-16.01] | 0.32 [0.23-0.44] | 1.62 [0.95-2.59] | 0.04 [0.03-0.07] |  | | -0.47 [-0.55 to -0.39] | -0.47 [-0.55 to -0.39] |
| Sahel Region | 33 [24.86-43.88] | 5.22 [3.97-6.89] | 4.49 [2.68-7.01] | 0.71 [0.43-1.1] |  | 48.44 [32.23-70.38] | 2.74 [1.87-3.92] | 6.64 [3.82-10.77] | 0.38 [0.21-0.61] |  | | -2.85 [-3.21 to -2.48] | -2.81 [-3.17 to -2.45] |

Table S2. The prevalence and years lived with disability (YLDs) of pelvic inflammatory disease correlated to Gonococcal infection in the GBD super regions. ASPR: age-standardized prevalence rates; EAPC: estimated annual percentage change

|  |  | 1990 |  |  |  |  | 2021 |  |  |  |  | | 1990-2021 |
| --- | --- | --- | --- | --- | --- | --- | --- | --- | --- | --- | --- | --- | --- |
|  | Prevalent cases No. *102 (95% UI) | ASPR per 100,000 No. (95% UI) | YLD cases No. *102 (95% UI) | Age-standardized YLD rate per 100,000 No. (95% UI) |  | Prevalent cases No. *102 (95% UI) | ASPR per 100,000 No.(95% UI) | YLD cases No. *102 (95% UI) | Age-standardized YLD rate per 100,000 No.(95% UI) |  | EAPC of ASPR No. (95% CI) | | EAPC of Age-standardized YLD rate No. (95% CI) |
| Global | 4722.43 [3547.59-6045.57] | 18.49 [13.92-23.85] | 641.43 [398.86-976.12] | 2.51 [1.57-3.84] |  | 7884.74 [5894.06-10325.44] | 19.49 [14.54-25.56] | 1072.11 [658.99-1657.63] | 2.65 [1.63-4.09] |  | -0.01 [-0.06 to 0.03] | | -0.01 [-0.05 to 0.04] |
| Socio-demographic index |  |  |  |  |  |  |  |  |  |  | |  |  |
| High SDI | 777.44 [565.56-1023.81] | 16.34 [11.94-21.46] | 106.52 [62.59-164.65] | 2.24 [1.32-3.42] |  | 982.91 [754.45-1257.61] | 18.25 [14.07-23.17] | 134.43 [84.41-201.66] | 2.5 [1.58-3.78] |  | | 0.04 [-0.09 to 0.17] | 0.04 [-0.09 to 0.17] |
| High-middle SDI | 806.55 [591.45-1068.6] | 14.66 [10.77-19.41] | 110.07 [66.39-167.93] | 2 [1.21-3.07] |  | 1115.94 [828.38-1448.47] | 15.58 [11.63-20.35] | 152.07 [93.13-231.59] | 2.12 [1.31-3.28] |  | | 0.3 [0.23 to 0.36] | 0.3 [0.24 to 0.36] |
| Middle SDI | 1222.24 [899.21-1594.56] | 15.15 [11.27-19.84] | 166.4 [101.6-254.61] | 2.06 [1.26-3.16] |  | 2274.2 [1700.69-2965.45] | 17.11 [12.86-22.34] | 309.37 [187.29-473.25] | 2.33 [1.41-3.56] |  | | 0.34 [0.31 to 0.38] | 0.35 [0.32 to 0.38] |
| Low-middle SDI | 1188.67 [921.64-1526.7] | 23.78 [18.39-30.14] | 160.33 [100.98-246.72] | 3.21 [2.06-4.89] |  | 2165.79 [1588.65-2863.47] | 21.89 [16.07-28.95] | 294 [175.29-460.27] | 2.97 [1.78-4.65] |  | | -0.53 [-0.63 to -0.43] | -0.51 [-0.61 to -0.41] |
| Low SDI | 723.62 [571.12-905.67] | 34.79 [27.48-43.37] | 97.58 [61.39-144.52] | 4.69 [2.97-6.94] |  | 1341.19 [974.41-1798.2] | 26.75 [19.61-35.59] | 181.59 [109.02-278.66] | 3.62 [2.18-5.59] |  | | -1.34 [-1.53 to -1.14] | -1.32 [-1.52 to -1.12] |
| Region |  |  |  |  |  |  |  |  |  |  | |  |  |
| Southeast Asia, East Asia, and Oceania | 1060.33 [779.71-1402.58] | 12.9 [9.52-17.09] | 144.75 [87.42-220.78] | 1.76 [1.06-2.72] |  | 1566.46 [1183.71-2016.7] | 13.01 [9.8-16.91] | 213.27 [131.67-324.17] | 1.77 [1.09-2.73] |  | | 0.11 [-0.01 to 0.24] | 0.11 [-0.01 to 0.24] |
| North Africa and Middle East | 166.06 [121.33-218.69] | 12.01 [8.74-15.81] | 22.59 [13.53-34.81] | 1.63 [0.98-2.51] |  | 393.69 [278.49-518.58] | 12.13 [8.68-15.93] | 53.69 [31.74-83.53] | 1.65 [0.99-2.56] |  | | 0.25 [0.15 to 0.35] | 0.26 [0.15 to 0.36] |
| Central Europe, Eastern Europe, and Central Asia | 405.01 [297.6-532.19] | 18.38 [13.5-24.09] | 55.15 [33.21-83.36] | 2.5 [1.51-3.78] |  | 413.84 [304.16-541.88] | 18.42 [13.52-24.16] | 56.39 [34.13-87.98] | 2.51 [1.51-3.85] |  | | -0.03 [-0.12 to 0.07] | -0.02 [-0.12 to 0.07] |
| Latin America and Caribbean | 251.38 [189.42-322.89] | 13.73 [10.26-17.67] | 34.31 [21.41-51.95] | 1.87 [1.15-2.84] |  | 674.87 [513.2-868.09] | 20.56 [15.61-26.29] | 92.01 [56.59-139.3] | 2.8 [1.72-4.25] |  | | 1.48 [1.01 to 1.95] | 1.48 [1.01 to 1.94] |
| High-income | 762.69 [559.84-1006.47] | 15.66 [11.49-20.62] | 104.54 [61.94-160.65] | 2.15 [1.27-3.27] |  | 956.84 [735.43-1223.2] | 18.31 [14.06-23.18] | 130.98 [82.27-196.37] | 2.51 [1.58-3.79] |  | | 0.2 [0.06 to 0.35] | 0.2 [0.06 to 0.34] |
| South Asia | 1232.64 [953.48-1580.47] | 26.18 [20.33-33.26] | 166.14 [105.1-257.89] | 3.53 [2.25-5.44] |  | 2314.54 [1716.22-3072.18] | 23.86 [17.67-31.71] | 313.98 [187.14-486.77] | 3.24 [1.93-5.03] |  | | -0.56 [-0.66 to -0.46] | -0.54 [-0.64 to -0.44] |
| Sub-Saharan Africa | 844.31 [653.73-1079.39] | 40.15 [31.26-50.91] | 113.95 [70.91-167.97] | 5.42 [3.4-8.02] |  | 1564.5 [1115.02-2135.26] | 29.76 [21.37-40.02] | 211.79 [128.23-319.84] | 4.03 [2.44-6.13] |  | | -1.59 [-1.81 to -1.37] | -1.58 [-1.8 to -1.36] |
| WHO region | 4700.83 [3531.93-6017.23] | 18.52 [13.93-23.88] | 638.49 [397.15-972.08] | 2.52 [1.57-3.85] |  | 7858.51 [5874.18-10290.85] | 19.52 [14.56-25.59] | 1068.51 [656.76-1651.31] | 2.65 [1.63-4.1] |  | | -0.01 [-0.06 to 0.03] | -0.01 [-0.05 to 0.03] |
| European Union | 182.73 [132.56-241.52] | 8.27 [5.94-10.93] | 25.01 [14.97-38.81] | 1.13 [0.68-1.76] |  | 210.41 [160.77-264.9] | 9.79 [7.45-12.36] | 28.76 [17.78-43.4] | 1.34 [0.83-2.05] |  | | 0.72 [0.52 to 0.92] | 0.71 [0.51 to 0.91] |
| World Bank Regions | 4716.91 [3543.33-6038.6] | 18.5 [13.92-23.85] | 640.68 [398.42-974.99] | 2.51 [1.57-3.84] |  | 7878.1 [5889.13-10316.82] | 19.5 [14.54-25.57] | 1071.2 [658.46-1656.26] | 2.65 [1.63-4.1] |  | | -0.01 [-0.06 to 0.03] | -0.01 [-0.05 to 0.04] |
| African Union | 909.89 [702.21-1163.15] | 33.45 [26-42.55] | 122.83 [76.42-180.8] | 4.52 [2.83-6.67] |  | 1710.05 [1220.27-2316.75] | 26.18 [18.81-35.21] | 231.71 [140.91-352.42] | 3.55 [2.14-5.46] |  | | -1.37 [-1.57 to -1.16] | -1.35 [-1.56 to -1.14] |
| Commonwealth | 1791.53 [1381.84-2323.01] | 27.13 [20.92-35] | 241.8 [152.26-373.46] | 3.66 [2.31-5.61] |  | 3329.68 [2447.34-4388.26] | 24.68 [18.14-32.68] | 451.45 [268.53-693.64] | 3.35 [2.01-5.18] |  | | -0.66 [-0.79 to -0.54] | -0.65 [-0.77 to -0.52] |
| G20 | 3055.93 [2274.91-3982.69] | 16.69 [12.47-21.76] | 415.83 [255.61-641.06] | 2.27 [1.4-3.48] |  | 4770.67 [3580.16-6205.96] | 18.38 [13.86-23.89] | 649.33 [395.8-990.8] | 2.5 [1.53-3.84] |  | | 0.19 [0.15 to 0.24] | 0.2 [0.15 to 0.24] |
| OECD Countries | 912.72 [667.42-1201.51] | 15.48 [11.31-20.33] | 125.08 [74.29-191.72] | 2.12 [1.26-3.24] |  | 1206.36 [930.24-1542.59] | 17.75 [13.7-22.58] | 164.98 [103.05-245.26] | 2.43 [1.52-3.65] |  | | 0.22 [0.1 to 0.35] | 0.22 [0.1 to 0.34] |
| Four World Regions | 4714.92 [3541.98-6035.96] | 18.5 [13.92-23.85] | 640.41 [398.21-974.71] | 2.51 [1.57-3.84] |  | 7876.97 [5888.26-10315.37] | 19.5 [14.54-25.57] | 1071.04 [658.37-1656.11] | 2.65 [1.63-4.1] |  | | -0.01 [-0.06 to 0.03] | -0.01 [-0.05 to 0.04] |
| Gulf Cooperation Council | 9.36 [6.73-12.59] | 11.63 [8.33-15.55] | 1.27 [0.73-2.07] | 1.58 [0.91-2.49] |  | 38.83 [27.13-52.64] | 11.55 [8.29-15.33] | 5.27 [2.95-8.57] | 1.57 [0.89-2.51] |  | | -0.04 [-0.08 to -0.01] | -0.03 [-0.07 to 0] |
| Organization of Islamic Cooperation | 1035.39 [798.58-1320.56] | 23.17 [17.94-29.41] | 140.01 [86.89-211.58] | 3.13 [1.97-4.71] |  | 1983.03 [1439.16-2661.65] | 19.93 [14.54-26.56] | 268.92 [160.64-411.64] | 2.7 [1.61-4.12] |  | | -0.82 [-1.01 to -0.63] | -0.81 [-0.99 to -0.62] |
| Nordic Region | 8.75 [6.46-11.49] | 7.24 [5.4-9.44] | 1.2 [0.72-1.86] | 0.99 [0.6-1.54] |  | 12.22 [8.87-16.19] | 9.43 [6.84-12.48] | 1.68 [0.99-2.65] | 1.3 [0.75-2.04] |  | | 0.82 [0.22 to 1.43] | 0.82 [0.22 to 1.43] |
| Health System Grouping Levels | 4718.51 [3544.63-6040.56] | 18.49 [13.92-23.85] | 640.9 [398.54-975.32] | 2.51 [1.57-3.84] |  | 7880.02 [5890.55-10319.33] | 19.5 [14.54-25.57] | 1071.46 [658.61-1656.66] | 2.65 [1.63-4.1] |  | | -0.01 [-0.06 to 0.03] | -0.01 [-0.05 to 0.04] |
| Association of Southeast Asian Nations | 168.2 [120.78-219.59] | 7.89 [5.74-10.29] | 23.01 [13.74-35.41] | 1.08 [0.64-1.67] |  | 284.96 [212.55-369.28] | 7.7 [5.76-9.98] | 38.8 [23.66-59.89] | 1.05 [0.64-1.62] |  | | -0.13 [-0.18 to -0.08] | -0.13 [-0.19 to -0.08] |
| Sahel Region | 303 [239.52-377.3] | 49.66 [39.39-61.47] | 40.84 [26.12-59.19] | 6.69 [4.3-9.71] |  | 563.49 [401.68-785.04] | 33.11 [23.54-45.61] | 76.16 [45.68-116.29] | 4.47 [2.71-6.88] |  | | -2.01 [-2.32 to -1.7] | -2 [-2.31 to -1.68] |

Table S3. The prevalence and years lived with disability (YLDs) of pelvic inflammatory disease correlated to other sexually transmitted infections in the GBD super regions. ASPR: age-standardized prevalence rates; EAPC: estimated annual percentage change
